# Effect of Additives during Interfacial Polymerization Reaction for Fabrication of Organic Solvent Nanofiltration (OSN) Membranes

**DOI:** 10.3390/polym13111716

**Published:** 2021-05-24

**Authors:** Su-Min Kim, Sena Hong, Bao-Tran Duy Nguyen, Hai-Yen Nguyen Thi, Sang-Hee Park, Jeong-F. Kim

**Affiliations:** 1 Department of Energy and Chemical Engineering, Incheon National University, Incheon 22012, Korea; 202121066@inu.ac.kr (S.-M.K.); sena.hong19@gmail.com (S.H.); bao.nguyen@inu.ac.kr (B.-T.D.N.); haiyen0107@inu.ac.kr (H.-Y.N.T.); 2 Department of Chemical Engineering, Changwon National University (CNU), Changwon 51140, Korea

**Keywords:** organic solvent nanofiltration, interfacial polymerization, thin film composite membranes, additives, surfactant

## Abstract

Thin film composite (TFC) membranes is the dominant type of desalination in the field of membrane technology. Most of the TFC membranes are fabricated via interfacial polymerization (IP) technique. The ingenious chemistry of reacting acyl chlorides with diamines at the interface between two immiscible phases was first suggested by Cadotte back in the 1980s, and is still the main chemistry employed now. Researchers have made incremental improvements by incorporating various organic and inorganic additives. However, most of the TFC membrane literature are focused on improving the water desalination performance. Recently, the application spectrum of membrane technology has been expanding from the aqueous environment to harsh solvent environments, now commonly known as Organic Solvent Nanofiltration (OSN) technology. In this work, some of the main additives widely used in the desalination TFC membranes were applied to OSN TFC membranes. It was found that tributyl phosphate (TBP) can improve the solubility of diamine monomer in the organic phase, and sodium dodecyl sulfate (SDS) surfactant can effectively stabilize the IP reaction interface. Employing both TBP and SDS exhibited synergistic effect that improved the membrane permeance and rejection in solvent environments.

## 1. Introduction

With the escalating global climate crisis, membrane technology has been gaining considerable attention as a new platform for the energy-efficient separation process. Particularly, chemical industries consume 10–15% of the total US energy consumption, mostly on distillation [1]. In comparison, membrane technology can perform high-precision separations without phase change while consuming approximately 90% less energy. One of the major advantages of membrane technology is that it is modular, and can be easily integrated with other separation technologies such as distillation, extraction, chromatography, recrystallization, and others.

The application spectrum of membrane technology has been expanding from simple water treatment to gas separations [2], artificial organs [3], and chemical separations in harsh organic media [4]. Particularly, organic solvent nanofiltration (OSN) technology has been attracting a lot of interest as it has the potential to perform difficult separation challenges posed in chemical industries.

Currently, most of the membrane markets employ polymeric membranes, although ceramic, zeolite, and metal-organic-framework membranes are also being developed actively. Better polymeric membranes with enhanced separation performance (permeance and selectivity) is always preferred. In the field of OSN, most of the early developments were focused on integrally skinned asymmetric (ISA) membranes, where solvent stability of base polymers was enhanced by heterogeneous crosslinking reaction. See-Toh et al. [5] first reported crosslinking of polyimide backbone with diamine to form crosslinked polyamide membranes. Polyimide is soluble in polar aprotic solvents such as *N,N*-dimethylformamide (DMF), *N*-methylpyrrolidone (NMP), and *N,N*-dimethylacetamide (DMAc). Polyimide membrane was first fabricated from DMF via phase inversion method; then, the membrane was cross-linked in diamine solution in heterogenous reaction. The crosslinked membranes became stable in harsh organic solvents, including polar aprotic solvents such as DMF, NMP and DMAc, while maintaining nanofiltration separation performance. This was a big breakthrough, as it opened new doors for membrane technology to enter fine chemical and pharmaceutical industries. Nowdays, OSN membranes are widely applied in such industries to recycle organic solvents [6] and homogeneous catalysts [7]. Valtcheva et al. [8,9] also reported similar strategy with polybenzimidazole (PBI) polymer, where alkylation chemistry was employed with dibromoxylene to fasten two imidazole rings which significantly improved the polymer solvent stability.

Similar to reverse osmosis (RO) membrane development, the trend in the field of OSN is shifting from ISA to thin film composite (TFC) membranes [10,11,12]. A TFC membrane is generally composed of three layers: a nonwoven support as the bottom layer, an ultrafiltration membrane as the middle layer, and a thin polymeric film on the top layer. The bottom layer acts as a mechanical support to improve the handleability, the middle layer acts as another support layer, and the top layer is the selective layer which performs the actual separation. The key advantage of TFC membranes is that each layer can be semi-independently optimized, and in particular, many interesting polymer chemistries can be applied to the top selective layer.

The top selective layer can be formed by several methods, such as solution-coating, lamination, vapor deposition, and interfacial polymerization (IP). Among different methods, the IP method is by far the most employed technique, and most of the RO membranes are commercialized with this method. The ingenious IP chemistry was first reported by Cadotte in 1980s [13], where trimesoyl chloride (TMC) was reacted with *m*-phenylenediamine (MPD) to form thin crosslinked polyamide films. The reaction proceeds at an interface between two liquid phases, generally at water-hexane or water-toluene interface. Apart from aforementioned TMC-MPD chemistry by Cadotte, membrane researchers have tested various types and permutations of chemicals in different compositions and conditions, for more than 40 years [14]. Remarkably, the TMC-MPD chemistry is still the most widely used IP pair both in current literature and in industry.

In addition, researchers have tried various additives to enhance reaction efficiency and membrane performance. Notably, use of acid scavengers and surfactants have shown positive effects in the membrane performance [15]. More recently, researchers incorporated nanoparticles to fabricate thin film nanocomposite (TFN) membranes [16], although the reported performance enhancements are questionable from solution-diffusion model perspectives. Every TFC membrane manufacturer has their own undisclosed recipe, and most of them certainly incorporate more than one additive to control their membrane performance.

It should be stressed that these developments were tailored towards aqueous applications involving desalination or other forms of water treatment. Many interesting reports on IP chemistry to prepare TFC membranes have not yet been tested for fabrication of OSN membranes. Therefore, in this work, some of the most widely employed additives and techniques to prepare RO membranes were used to fabricate TFC membranes for OSN applications. Three different additives were tested: tributyl phosphate (TBP) additive in the organic phase, triethylamine (TEA) additive in the aqueous phase, and sodium dodecylsulfate (SDS) surfactant in the aqueous phase. The effect of each additive in the OSN performance was systematically investigated. It was found that appropriate selection of additives certainly improves the TFC-OSN membrane performance.

## 2. Experimental

### 2.1. Materials

Two different commercial membranes were used as supports for TFC membranes: JQM-PS-500 membrane (polysulfone, average pore size 0.05 μm, JiaQuan, Guangdong, China), and M-M2540PS20 membrane (polysulfone, MWCO 20,000 Da, Applied Membranes, Vista, CA, USA). In this work, these two support membranes are annotated as PSf1 and PSf2, respectively. *m*-Phenylenediamine (MPD, 98.0%), trimesoyl chloride (TMC, 98.0%), triethylamine (TEA, 99.0%), and sodium dodecyl sulfate (SDS) were purchased from Sejin CI (South Korea). Potassium carbonate (K_2_CO_3_, 99.5%), tributyl phosphate (TBP, 99.0%), ethanol (EtOH, 99.5%), isopropyl alcohol (IPA, 99.5%), and *n*-hexane (95.0%) were purchased from Samchun Chemicals (Seoul, South Korea). Sodium Chloride (NaCl, 99.05%) was purchased from Daejung Chemicals (Goryeong, South Korea) and polypropylene glycol (PPG, 425 Da, 725 Da, 1000 Da) was purchased from Sigma-Aldrich (Seoul, South Korea).

### 2.2. Membrane Fabrication

Prior to interfacial polymerization (IP) reaction, the support membranes (PSf1 or PSf2) were immersed in EtOH for 30 min to remove any pore preservatives, followed by DI water for 30 min. The washed support membrane was then immersed in the 3 wt% aqueous MPD solution containing SDS (0–2 wt%) or TEA (0–0.9 wt%) for 5 min. The membrane surface was wiped off using a rubber roller to remove excess aqueous solution, then it was affixed with a silicone gasket. An organic TMC solution (0.23 wt%) containing TBP (0–0.6 wt%) in *n*-hexane was then poured onto the MPD-impregnated support surface and allowed to react at room temperature for 3 min. After IP reaction, the membrane was exposed to different set of post-treatments such as hexane rinse, air dry, and heat treatment (60 °C for 1 min). The fabricated TFC membranes were kept in DI water.

### 2.3. Membrane Characterization

The morphologies of TFC membranes were analyzed using FE-SEM (field emission scanning electron microscopy, JSM-7800F, Japan). The chemical nature of the membranes were characterized using ATR-FTIR (attenuated total reflectance—fourier transform infrared spectroscopy, IR Tracer-100, Shimadzu, Kyoto, Japan)

### 2.4. Membrane Performance Evaluation

**Figure 1:**
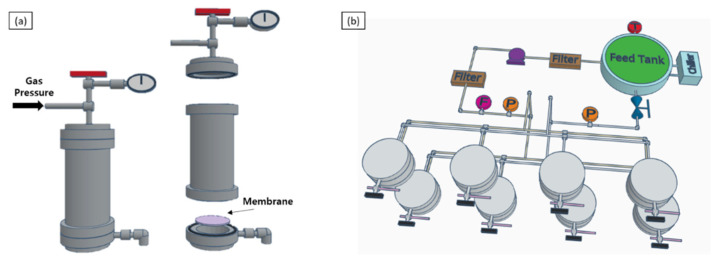
Schematics of (**a**) dead-end cell apparatus, and (**b**) in-house cross-flow system with 8 membrane cells in parallel configuration.

The permeance and rejection performance of the membranes were characterized using both dead-end cell and in-house crossflow system, as shown in Figure 1.

The membrane permeance was calculated using the following equation.

(1) $$\mathrm{Permeance}\;=\frac{V}{A\;t\;\Delta P}\equiv \;\left[\frac{L}{m^{2}\;h\;\mathrm{bar}}\right]$$

The membranes with effective surface area of 16.6 cm^2^ each were tested in EtOH and IPA solvent containing 2 g·L^−1^ PPG oligomers (MW range between 350–1200 Da). The solution was circulated in crossflow at 30 bar using a high-pressure diaphragm pump (G03, HydraCell, Wanner Engineering Inc, Minneapolis, MN, USA) at 150 L·h^−1^. The solution temperature was maintained at 30 °C using a double-jacketed feed tank and a chiller. Permeate samples were collected after 6 h to condition and stabilize the membrane performance. The samples were analyzed using an HPLC (high performance liquid chromatography, YL9100 Plus, YoungIn Chromass Inc, Anyang, South Korea) equipped with an ELSD (evaporative light scattering detector). The mobile phase was a mixture of water and acetonitrile (gradient), and the stationary phase was a C18 column (4.6 mm × 150 mm, 5 μm). The column separates PPG oligomer based on the MW, and relative concentration of each solute of permeate and retentate can be calculated from the calibration data. The observed rejection value of each solute MW can be calculated using the following equation.

(2) $$\mathrm{Rejection},\;R\;\left(\%\right)=\left(1-\frac{C_{p}}{C_{R}}\right)\times 100\%$$

## 3. Results and Discussion

It has been reported many times that the surface morphology and the chemical nature of the support membrane affects the IP reaction significantly [17,18,19,20]. In this work, two different commercial polysulfone membranes (PSf1, PSf2) were used as supports for IP reaction. According to the manufacturer specifications, PSf1 membranes have average pore size of 0.05 μm, whereas PSf2 membranes have molecular weight cut off (MWCO) value of 20,000 Da. Note that the MWCO is the molecular weight of a solute that is 90% rejected by the membrane; hence, lower the MWCO, the smaller the pore size. Generally, microfiltration membranes are characterized using the average pore size value, and ultrafiltration membranes are described using the concept of MWCO.

**Figure 2:**
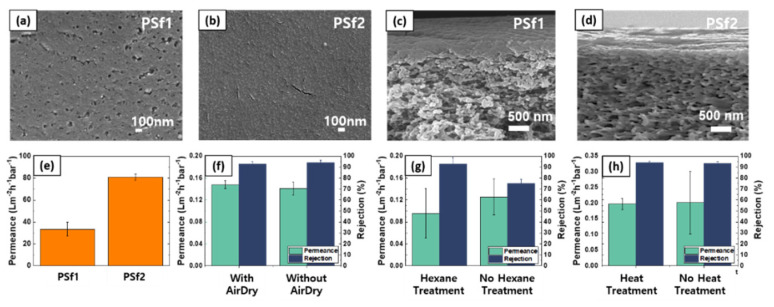
Polysulfone support membrane characterization and effect of post interfacial polymerization treatment. (**a**–**d**) surface and cross-sectional SEM images of PSf1 and PSf2, respectively; (**e**) pure water permeance of PSf1 and PSf2; (**f**–**h**) effects of air dry, hexane rinse, and heat treatment on TFC membrane water permeance and NaCl rejection, respectively. The membranes were tested with dead-end cell apparatus.

It can be seen in Figure 2 that PSf1 membrane has lower water permeance than that of PSf2. On the other hand, the surface pores are clearly visible in PSf1, whereas there are no micro-scale pores in PSf2. Hence, clearly, the pore size alone cannot be the sole factor to estimate the permeance. In this particular case, PSf2 may exhibit very high pore connectivity beneath the surface, but it is difficult to conclude from the SEM images. Apart from the membrane permeance, PSf1 membrane is expected to exhibit higher surface roughness, which can have an adverse effect during IP reaction. Therefore, PSf2 membranes were used as supports for IP reaction to fabricate thin film composite (TFC) membranes.

The density of the synthesized thin polymer film (e.g., polyamide) is also highly dependent on the post-reaction treatments [21,22,23,24,25]. The effects of three different post-IP treatments are summarized in Figure 2f–h. It was determined that with the IP condition employed in this work (3 wt% MPD for 5 min, 0.23 wt% TMC for 3 min), both air dry and heat treatment did not affect the membrane performance. In comparison, rinsing the membrane with hexane to remove unreacted monomers improved the NaCl rejection. It should be noted that the effects of these post-treatments vary among the reported literature [21,25], indicating that IP reaction is highly sensitive to other conditions such as support membrane morphology. In this work, only the hexane rinsing step was employed for preparation of TFC membranes.

Many additives have been reported by literatures to improve the performance of TFC membranes for desalination (reverse osmosis, RO). Researchers have tested various permutations of monomer compositions and additives, but their effects have not been explicitly tested in solvent filtrations. We have tested three well-known additives in the IP literature: TBP in the organic phase, each TEA and SDS surfactant in the aqueous phase. The exact mechanism and purpose of each additive are different.

**Figure 3:**
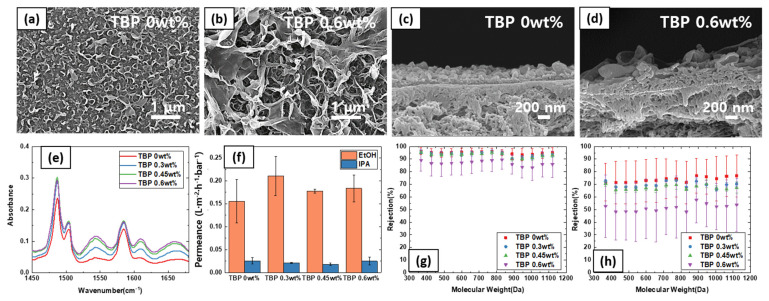
Effect of TBP additives in the organic phase on (**a**,**b**) surface SEM images, (**c**,**d**) cross-sectional SEM images, (**e**) FTIR spectra (**f**) solvent permeance, (**g**) PPG rejection profile in EtOH, and (**h**) PPG rejection profile in IPA.

Effects of TBP in the organic phase on TFC membrane morphology and performance are summarized in Figure 3. The use of TBP in the IP reaction was first proposed by Kim et al. [26]. The idea for using the TBP additive during an IP reaction is to improve the solubility of both TMC and MPD in the organic phase. The IP reaction proceeds in the organic-aqueous interface, but it mostly occurs in the organic layer, as MPD diffuses into the organic layer and reacts with the TMC (TMC is not soluble in water). The IP reaction self-terminates as the film grows thicker and hinders further MPD diffusion into the organic phase.

As shown in Figure 3a–d, incorporating TBP (0–0.6 wt%) in the organic phase resulted in a drastic difference in the membrane morphology. Compared to the typical ridge-and-valley surface morphology of the TFC membrane, employing TBP additive resulted in thicker film and the surface structure became more distinct. FTIR analysis shown in Figure 3d clearly show a qualitative trend towards thicker films. Four characteristic polyamide peaks can be observed at 1487 cm^−1^ (C–C stretching of benzene ring), 1543 cm^−1^ (N–H in-plane bending), 1608 cm^−1^ (H-bonded C=O stretching), and 1670 cm^−1^ (C=O stretching of the amide bond), confirming that polyamide films were formed by the IP reaction. Importantly, the peak intensity progressively became bigger with higher TBP content, indicating the TFC layer accordingly became thicker. Hence, as expected, TBP promotes IP reaction by improving the solubility of MPD in the organic phase and stabilizing the reaction surface.

On the other hand, the addition of TBP did not affect the OSN performance in EtOH and IPA. There was a slight improvement in EtOH permeance with the addition of 0.3 wt% TBP, but the results were not statistically significant. Interestingly, as the TBP content increased to 0.6 wt%, a noticeable drop in the membrane rejection of PPG oligomer was observed for both EtOH and IPA solvent. As TBP is also soluble in the aqueous phase, higher TBP content can destabilize the aqueous-organic phase interface, leading to loose TFC layer or defects.

Also, it can be seen that the PPG rejection curve in IPA is generally lower than those of EtOH. This is due to the fact that the permeation proceeds by the solution-diffusion mechanism. The rejection is determined by the relative permeance of solvent and PPG solute. The permeance of IPA solvent is generally lower than that of EtOH, as IPA is larger, more hydrophobic, and more viscous. These factors lower the diffusivity of IPA compared to that of EtOH. Therefore, in accordance with the solution-diffusion mode, the solute rejection profile in IPA is lower than EtOH for TFC membranes.

Aqueous-soluble base compound is an important class of additive used for IP reaction via TMC-MPD chemistry. The byproduct of TMC-MPD reaction is HCl acid, and the solution progressively becomes more acidic [27], known to adversely affect the efficiency of IP reaction. Hence, addition of TEA as a mild base can neutralize the formed acids to maintain constant solution pH. Many works have been reported that show the positive effect of TEA [21,28].

**Figure 4:**
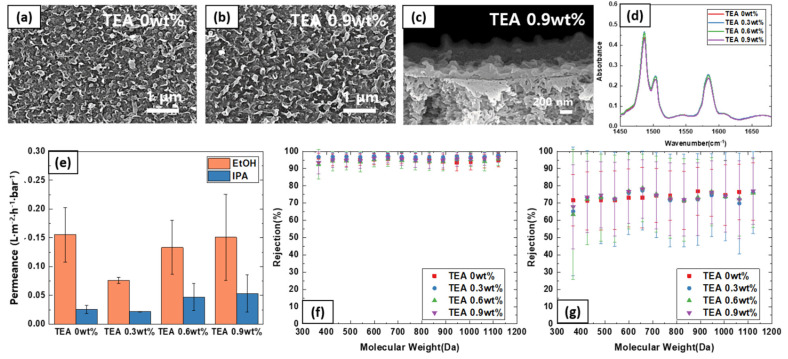
Effect of TEA additives in the aqueous phase on (**a**,**b**) surface SEM images, (**c**) cross-sectional image; (**d**) FTIR spectra. (**e**) solvent permeance (**f**) PPG rejection profile in EtOH, and (**g**) PPG rejection profile in IPA.

The data for TFC membranes fabricated using TEA additive are summarized in Figure 4. Compared to the TBP results, there was no noticeable difference in the surface and cross-sectional morphology of TFC membranes. There was also no difference in the membrane thickness nor the chemistry, as indicated by the FTIR data. As for the permeance and rejection trend, no statistically significant difference was observed for both EtOH and IPA, except a slight drop in the EtOH permeance with 0.3 wt% TEA addition.

The SDS surfactant is another common additive used during an IP reaction. As water aqueous phase and hexane organic phase are immiscible, a slight perturbation in the interface can destabilize the reaction interface, leading to undesired defects. This is one of the main reasons why the reproducibility of IP reaction in laboratory, particularly for unexperienced researchers, is low. To overcome this, addition of surfactant (e.g., SDS) can enlarge and effectively stabilize the reaction interface, improving the reproducibility of IP reaction quite significantly. Particularly, the use of SDS has been reported many times with positive effects [28,29,30].

**Figure 5:**
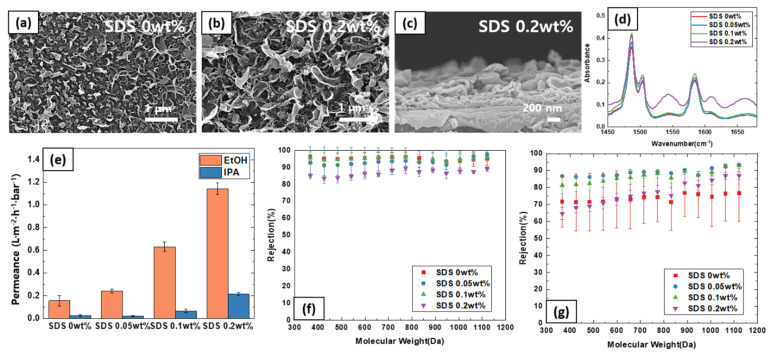
Effect of SDS additives in the aqueous phase on (**a**,**b**) surface SEM images, (**c**) cross-sectional image; (**d**) FTIR spectra. (**e**) Solvent permeance (**f**) PPG rejection profile in EtOH, and (**g**) PPG rejection profile in IPA.

The effects of SDS in TFC membrane fabrication are summarized in Figure 5. It can be seen that, similar to TBP additive, the surface morphology become more distinct with the addition of SDS. This is due to the fact that the reaction interface is stabilized with surfactant. Naturally, the polyamide thin film progressively thickens with SDS concentration. FTIR analysis confirms the presence of polyamide layer and the thickness. Such trend was similar to that of TBP additive.

In contrast to the TBP additive, interestingly, a drastic improvement in solvent permeance was observed with SDS additive. Notably, the EtOH permeance improved nearly by 6-fold when 0.2 wt% SDS was added to the aqueous layer. There was also statistically significant impact on the PPG rejection trend. Particularly, two opposing trends can be deduced. With the addition of SDS, the solvent permeance was enhanced, which generally leads to higher rejection (relative permeance of solvent to solute increases, leading to higher observed rejection). However, as the SDS content becomes excessive (>0.2 wt%), the film becomes too loose and the film selectivity deteriorates. It can be seen that the rejection goes down for both EtOH and IPA with 0.2 wt% SDS content. 

**Figure 6:**
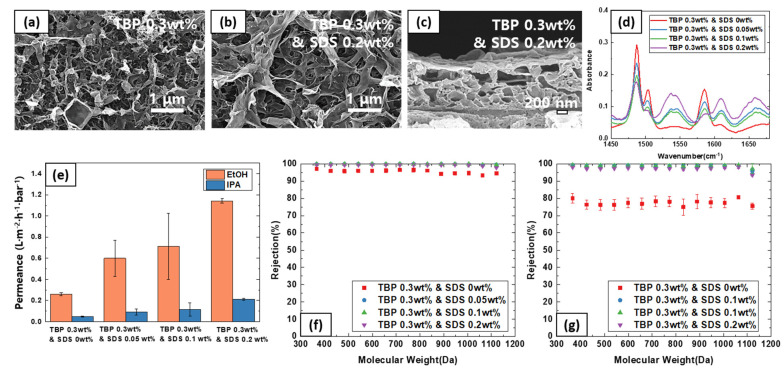
Effect of SDS and TBP additives on (**a**,**b**) surface SEM images, (**c**) cross-sectional image, (**d**) FTIR spectra, (**e**) solvent permeance, (**f**) PPG rejection profile in EtOH, and (**g**) PPG rejection profile in IPA.

Since TBP in the organic phase and SDS additive in the aqueous phase both affected the membrane morphology and performance, two additives were used in tandem to investigate the synergistic effect during the IP reaction. The results are summarized in Figure 6. TBP content was fixed at 0.3 wt% and SDS content was varied from 0 wt% to 0.2 wt%. As expected, the film thickness increased accordingly, and the surface morphology became distinct. FTIR analysis confirmed the thickening effect as well as the successful formation of the polyamide layer.

As for the membrane performance, there was a significant improvement in both permeance and rejection profile. The permeance improved as expected from Figure 5 data. Interestingly, the membrane rejection increased significantly for both EtOH and IPA solvent, reaching near 100% even for small molecular weight PPG oligomers. Such simultaneously improvement can be attributed to two effects. Addition of TBP increases the MPD solubility, and addition of SDS stabilizes the reaction surface. Therefore, dense polaymide film was able to form under relatively stable reaction environment. Hence, there was a clear synergistic effect by employing both TBP and SDS simultaneously.

## 4. Conclusions

Various types of additives were employed during preparation of thin film composite membranes via IP reaction for OSN applications. It was found that employing TBP additive in the organic phase enhances the MPD solubility and results in thicker membranes. Although noticeable difference was observed in the film morphology, there was no significant change in the membrane performance in organic solvents. Since TBP is also soluble with the aqueous phase, increasing the TBP content above certain threshold (>0.6 wt%) resulted in deterioration of the membrane selectivity. TEA additive was employed as the neutralizing agent to scavenge the acid compounds that form during the IP reaction. Contrary to other literature reports where TEA showed positive effects in desalination applications, there was no observable improvement in membrane performance in solvent environments. On the other hand, employing SDS surfactant as an additive resulted in significant improvement in membrane permeance. It was found that the use of SDS stabilizes the reaction interface by enlarging it, resulting in reproducible results with thicker films. Interestingly, employing both TBP and SDS simultaneously improved both membrane permeance and rejection in tandem. The permeance improved by near 6-fold, and the rejection of PPG oligomer reached near 100% across all molecular weight range. Such synergistic effect was induced as TBP enhances the MPD solubility while SDS stabilizes the reaction interface. Hence, employing both TBP and SDS during IP can reproducibly enhance the performance of TFC-OSN membranes. Additionally, several post-treatments were tested in this work, and found that rinsing the formed membrane with hexane removed unreacted monomers and improved the film density. Apart from these additives employed in this work, there are still many unique additives and combination of additives developed for desalination membranes, yet to be tested for OSN applications.

## Data Availability

The data presented in this study are available on request from the corresponding author.
